# Supplementary material for: A Likelihood Approach for Real-Time Calibration of Stochastic Compartmental Epidemic Models
Source: PLoS Comput Biol. 2017 Jan 17;13(1):e1005257. doi: 10.1371/journal.pcbi.1005257 (PMC5240920; doi:10.1371/journal.pcbi.1005257)
Supplement: S1 File — (TAR.GZ) [file pcbi.1005257.s014.tar.gz › HSPH_Online-SI-Revision/IPoi09/n100-severe/n100-severe_BENCH_table-4.pdf]

1	R0	{1.65278, 9.06793}	{1.87941, 9.82867}	{{1.87927, 9.84456}, {1.87927, 9.84456}}	{13.7118, 8.38936}	inf	4882.	9002.61	{2565., 13 025.}	102.295	0.327155	newly	4385.	6987.33	{2442., 9385.}	76.7086	0.273533	Reff	1.41083	1.39129	{1.0874, 1.77204}	15.6598	ar	66 342.	75 992.3	{75 584., 76 490.}	14.5463
2	R0	{1.4321, 3.80644}	{1.66021, 4.3883}	{{1.64953, 4.30394}, {1.64953, 4.30394}}	{15.9282, 15.5041}	inf	1776.	5200.83	{848., 8375.}	208.644	0.472708	newly	4438.	9935.65	{2325., 14 558.}	138.276	0.37864	Reff	1.34796	1.39752	{1.1552, 1.61598}	11.8751	ar	54 217.	67 715.6	{66 086., 67 783.}	24.8973
3	R0	{1.68388, 4.76028}	{2.28084, 7.51245}	{{2.21381, 7.10188}, {2.2769, 7.49313}}	{35.4513, 57.8154}	inf	2116.	5534.11	{288., 10 408.}	196.332	0.526666	newly	4947.	7559.67	{529., 13 573.}	95.6542	0.418279	Reff	1.59182	2.04089	{1.8092, 2.2682}	28.5998	ar	68 299.	85 895.5	{84 812., 86 190.}	25.764
4	R0	{1.66014, 6.95383}	{1.53501, 4.02511}	{{1.53501, 4.02511}, {1.53501, 4.02511}}	{7.53732, 42.1166}	inf	3294.	5895.58	{3891., 6988.}	85.2704	0.27834	newly	4326.	10 279.8	{8275., 11 856.}	142.183	0.38904	Reff	1.51479	1.0782	{0.903983, 1.34088}	28.8222	ar	66 820.	60 554.2	{60 065., 61 260.}	9.37714
5	R0	{1.69984, 11.2038}	{1.67405, 7.28757}	{{1.67405, 7.28757}, {1.67405, 7.28757}}	{1.5172, 34.9545}	inf	6557.	8005.37	{5252., 9595.}	31.3727	0.125637	newly	4410.	7491.03	{5584., 9109.}	72.3851	0.231814	Reff	1.38564	0.994578	{0.738574, 1.40662}	29.4939	ar	68 462.	67 952.9	{67 276., 68 619.}	0.82393
6	R0	{1.65876, 6.66217}	{1.43579, 3.38828}	{{1.43579, 3.38828}, {1.43579, 3.38828}}	{13.442, 49.1415}	inf	2507.	4262.04	{2683., 4972.}	79.683	0.294267	newly	3575.	8488.91	{6723., 10 364.}	145.272	0.415838	Reff	1.55026	1.02925	{0.870778, 1.27524}	33.6077	ar	67 105.	53 839.5	{53 200., 54 612.}	19.7682
7	R0	{1.69966, 5.89996}	{1.61691, 4.41442}	{{1.61691, 4.41442}, {1.61691, 4.41442}}	{4.86862, 25.1788}	inf	2040.	3929.11	{306., 7269.}	129.787	0.439603	newly	3565.	7684.05	{757., 12 868.}	145.386	0.440279	Reff	1.61145	1.41066	{1.20617, 1.60404}	12.4601	ar	69 396.	65 101.2	{64 388., 65 743.}	6.18888
8	R0	{1.53428, 6.3734}	{1.54465, 5.30296}	{{1.54465, 5.30296}, {1.54465, 5.30296}}	{0.675887, 16.7954}	inf	2925.	4556.5	{1501., 7240.}	80.2051	0.272137	newly	3946.	6569.32	{2813., 8989.}	80.8803	0.256181	Reff	1.38868	1.24821	{0.930991, 1.4731}	13.2749	ar	59 855.	61 243.5	{60 362., 61 965.}	2.33087
9	R0	{1.58939, 3.48608}	{2.75382, 12.2217}	{{1.84836, 6.13642}, {2.91762, 13.2975}}	{73.2625, 250.587}	inf	458.	1733.09	{330., 3875.}	291.068	0.509893	newly	1645.	1663.58	{296., 3233.}	60.421	0.32553	Reff	1.56577	2.67616	{1.82328, 2.87477}	70.9166	ar	62 575.	90 984.5	{75 331., 93 557.}	45.4007
10	R0	{1.58528, 5.5593}	{2.70517, 19.3007}	{{2.53519, 17.5902}, {2.73517, 19.6025}}	{70.6432, 247.178}	inf	2476.	5944.13	{734., 13 431.}	171.137	0.474166	newly	4136.	2705.24	{408., 5681.}	50.7281	0.4364	Reff	1.4648	2.43247	{2.10083, 2.69212}	66.0616	ar	63 039.	91 569.5	{89 852., 92 057.}	45.2584
11	R0	{1.58305, 4.62721}	{1.81939, 5.69469}	{{1.81768, 5.68021}, {1.81768, 5.68021}}	{14.9292, 23.2535}	inf	1827.	4086.33	{940., 9464.}	145.49	0.393132	newly	3948.	6751.61	{1925., 13 442.}	95.8207	0.331549	Reff	1.50095	1.63055	{1.39601, 1.77789}	11.7039	ar	63 222.	73 815.4	{73 248., 74 350.}	16.756
12	R0	{1.70234, 9.34626}	{2.2179, 13.788}	{{2.18932, 13.5121}, {2.32014, 14.7828}}	{30.2853, 47.5243}	inf	5054.	8399.81	{1526., 16 670.}	99.9685	0.354695	newly	4443.	4952.28	{1061., 8985.}	56.6242	0.295042	Reff	1.46651	1.82323	{1.36933, 2.14595}	27.7466	ar	68 213.	84 759.5	{83 922., 86 713.}	24.2571
13	R0	{1.68556, 9.74954}	{1.85533, 9.28761}	{{1.85533, 9.28761}, {1.85533, 9.28761}}	{10.072, 4.73797}	inf	5632.	10 890.	{6644., 12 768.}	97.4563	0.294128	newly	4572.	8290.38	{5967., 9448.}	84.454	0.265644	Reff	1.41078	1.12879	{0.78191, 1.5466}	24.4068	ar	68 426.	75 291.3	{74 879., 75 728.}	10.0332
14	R0	{1.59876, 9.78897}	{1.68297, 7.5059}	{{1.68297, 7.5059}, {1.68297, 7.5059}}	{5.26721, 23.3229}	inf	4188.	8082.66	{5518., 9591.}	94.0362	0.280209	newly	3466.	7366.26	{5673., 8643.}	112.529	0.320862	Reff	1.39393	1.00679	{0.747306, 1.41799}	28.8127	ar	63 553.	68 311.3	{67 696., 68 803.}	7.48716
15	R0	{1.43577, 5.12819}	{1.9682, 10.257}	{{1.96658, 10.2409}, {1.96658, 10.2409}}	{37.0836, 100.012}	inf	1915.	9449.89	{3519., 14 436.}	401.181	0.698374	newly	3233.	7212.09	{3135., 10 051.}	132.548	0.40088	Reff	1.33374	1.48563	{1.10724, 1.81922}	19.0019	ar	53 259.	78 858.	{78 409., 79 253.}	48.0652
16	R0	{1.58866, 8.73191}	{1.89662, 10.0711}	{{1.87927, 9.84456}, {1.98131, 11.1772}}	{19.3847, 15.3368}	inf	3460.	8295.73	{1690., 13 123.}	160.475	0.446418	newly	3246.	6367.24	{1510., 9269.}	117.635	0.382093	Reff	1.43615	1.45708	{1.05769, 1.84108}	16.1559	ar	64 349.	76 645.9	{75 650., 79 249.}	19.1097
17	R0	{1.43012, 3.8163}	{1.37363, 2.81014}	{{1.37363, 2.81014}, {1.37363, 2.81014}}	{3.95002, 26.3648}	inf	970.	2598.68	{926., 3975.}	179.686	0.476664	newly	2605.	7264.91	{3191., 9823.}	187.828	0.496411	Reff	1.37832	1.17807	{1.0312, 1.31437}	14.5289	ar	53 353.	48 965.7	{48 188., 49 769.}	8.2231
18	R0	{1.47965, 5.16472}	{1.47608, 3.83841}	{{1.47608, 3.83841}, {1.47608, 3.83841}}	{0.241273, 25.6802}	inf	2352.	5023.69	{2213., 5944.}	117.9	0.328573	newly	4058.	9171.77	{5517., 10 781.}	126.231	0.34342	Reff	1.36212	1.07091	{0.858739, 1.36137}	21.7049	ar	56 086.	56 853.7	{56 175., 57 621.}	1.44414
19	R0	{1.57191, 5.2314}	{2.53075, 11.6597}	{{2.53075, 11.6597}, {2.53075, 11.6597}}	{60.9984, 122.879}	inf	3247.	8676.48	{853., 15 787.}	190.861	0.484994	newly	5657.	6745.21	{827., 11 501.}	59.8553	0.315675	Reff	1.41239	2.12683	{1.79843, 2.49565}	51.1121	ar	63 119.	89 653.4	{89 455., 89 835.}	42.0387
20	R0	{1.54435, 5.32911}	{2.89414, 17.9787}	{{2.90165, 18.0404}, {2.90165, 18.0404}}	{87.4017, 237.368}	inf	2562.	7857.95	{1149., 14 782.}	227.159	0.517331	newly	4511.	3977.9	{651., 7355.}	46.7911	0.321704	Reff	1.42398	2.51913	{2.14885, 2.85041}	76.9075	ar	61 870.	93 157.9	{93 120., 93 533.}	50.5704
21	R0	{1.57932, 4.90031}	{1.61691, 4.41442}	{{1.61691, 4.41442}, {1.61691, 4.41442}}	{2.38014, 9.9155}	inf	1638.	5674.05	{682., 8372.}	263.324	0.546418	newly	3321.	9766.41	{1816., 13 233.}	207.411	0.479729	Reff	1.50468	1.2582	{0.988757, 1.58572}	18.2136	ar	63 595.	65 101.2	{64 388., 65 743.}	2.36837
22	R0	{1.47044, 4.07694}	{1.5042, 4.74114}	{{1.50982, 4.79544}, {1.50982, 4.79544}}	{2.68558, 16.4882}	inf	941.	3943.3	{820., 6283.}	324.325	0.560978	newly	2369.	6548.7	{1677., 9434.}	186.609	0.431873	Reff	1.4165	1.25757	{1.05734, 1.46656}	12.6549	ar	55 895.	58 358.1	{54 119., 59 695.}	5.42446
23	R0	{1.64997, 3.34546}	{1.4031, 2.55684}	{{1.4031, 2.55684}, {1.4031, 2.55684}}	{14.9621, 23.5728}	inf	758.	1864.69	{137., 3877.}	177.938	0.522396	newly	3196.	6931.73	{729., 12 113.}	147.399	0.477878	Reff	1.61331	1.29859	{1.17099, 1.39693}	19.5077	ar	66 404.	51 398.2	{50 618., 52 249.}	22.5977
24	R0	{1.67405, 4.50254}	{1.4023, 2.07266}	{{1.4023, 2.07266}, {1.4023, 2.07266}}	{16.2331, 53.9669}	inf	1073.	1835.48	{359., 3998.}	98.6262	0.321311	newly	2825.	8954.63	{2254., 15 217.}	226.937	0.484	Reff	1.63156	1.29718	{1.15691, 1.38369}	20.4945	ar	68 017.	51 336.4	{50 349., 52 433.}	24.5241
25	R0	{1.50663, 3.1286}	{1.63789, 3.29506}	{{1.51531, 2.72346}, {1.65456, 3.37285}}	{8.71187, 8.26365}	inf	639.	1708.29	{152., 3331.}	188.134	0.522541	newly	2373.	6257.47	{689., 11 338.}	182.238	0.532414	Reff	1.47669	1.56233	{1.4763, 1.64726}	6.86467	ar	58 823.	64 597.1	{59 391., 67 389.}	14.2637
26	R0	{1.69822, 6.34992}	{2.79408, 15.842}	{{2.78517, 15.7559}, {2.78517, 15.7559}}	{64.5301, 149.483}	inf	2762.	6807.18	{1716., 14 211.}	168.841	0.432256	newly	4173.	3960.67	{1113., 7789.}	54.6993	0.345845	Reff	1.57542	2.4764	{2.06871, 2.70774}	57.1896	ar	69 816.	92 413.9	{92 146., 92 566.}	32.3677
27	R0	{1.55869, 4.92988}	{2.55533, 12.9697}	{{2.34121, 11.1899}, {2.63407, 13.6136}}	{63.9411, 163.083}	inf	1917.	5140.37	{687., 10 529.}	195.842	0.485579	newly	3834.	3854.72	{551., 7301.}	58.7051	0.360249	Reff	1.46882	2.31834	{2.0315, 2.60262}	57.8374	ar	62 344.	89 688.	{86 931., 90 993.}	43.8599
28	R0	{1.46085, 6.06899}	{1.65865, 5.3704}	{{1.65865, 5.3704}, {1.65865, 5.3704}}	{13.5401, 11.5108}	inf	3200.	7484.94	{4919., 9219.}	140.844	0.380764	newly	4410.	10 083.2	{8299., 11 744.}	132.225	0.362388	Reff	1.29135	1.15316	{0.906071, 1.42433}	17.0372	ar	55 484.	67 241.1	{66 639., 67 866.}	21.1901
29	R0	{1.63017, 6.25164}	{1.78005, 6.14025}	{{1.74092, 5.83416}, {1.74092, 5.83416}}	{9.1943, 10.7728}	inf	3520.	7526.19	{4073., 11 331.}	122.366	0.349896	newly	5090.	9636.12	{6259., 12 744.}	97.8431	0.31679	Reff	1.47729	1.43165	{1.13558, 1.67567}	11.8156	ar	66 231.	72 869.	{70 491., 82 648.}	10.0225
30	R0	{1.45173, 5.68165}	{2.8306, 19.6799}	{{2.56757, 17.005}, {2.86095, 19.9929}}	{94.9808, 246.377}	inf	2741.	9160.9	{2403., 15 769.}	248.555	0.531034	newly	3975.	4058.33	{1156., 6800.}	46.9075	0.276739	Reff	1.31862	2.3911	{2.04592, 2.7514}	81.333	ar	55 280.	92 623.7	{90 240., 93 174.}	67.5537
31	R0	{1.55808, 2.77316}	{1.57454, 2.99965}	{{1.51531, 2.72346}, {1.65456, 3.37285}}	{4.13119, 10.1746}	inf	583.	1584.64	{390., 3198.}	187.491	0.426456	newly	3043.	6148.26	{1875., 10 924.}	119.483	0.342855	Reff	1.52792	1.51606	{1.42348, 1.61745}	5.27996	ar	60 940.	63 410.1	{58 872., 67 181.}	6.61138
32	R0	{1.56797, 3.90327}	{2.09497, 7.14772}	{{2.05986, 6.93093}, {2.05986, 6.93093}}	{33.6103, 83.1213}	inf	984.	3031.71	{648., 6802.}	227.258	0.44455	newly	2775.	4515.24	{1088., 9339.}	100.121	0.333423	Reff	1.52355	1.9778	{1.79776, 2.0488}	29.8151	ar	62 228.	81 977.3	{80 860., 81 662.}	31.737
33	R0	{1.70027, 5.43261}	{1.54098, 3.31039}	{{1.54098, 3.31039}, {1.54098, 3.31039}}	{9.36851, 39.0645}	inf	2721.	4153.32	{1500., 6808.}	82.3298	0.307553	newly	5236.	10 596.4	{5176., 14 792.}	113.701	0.333887	Reff	1.58887	1.30411	{1.10186, 1.47224}	17.9224	ar	68 798.	60 965.5	{60 473., 61 629.}	11.3848
34	R0	{1.61668, 6.86192}	{2.41197, 16.4137}	{{2.41197, 16.4137}, {2.41197, 16.4137}}	{49.1928, 139.2}	inf	3615.	8138.3	{1100., 16 134.}	153.855	0.454666	newly	4645.	4099.78	{663., 7744.}	51.2899	0.372473	Reff	1.44922	2.03355	{1.59465, 2.35249}	40.9762	ar	64 693.	88 027.5	{87 797., 88 257.}	36.0696
35	R0	{1.55955, 3.45071}	{1.4804, 3.12918}	{{1.4804, 3.12918}, {1.4804, 3.12918}}	{5.07518, 9.31779}	inf	643.	1304.21	{89., 3269.}	148.442	0.490964	newly	2408.	4247.42	{341., 9922.}	121.012	0.448434	Reff	1.53037	1.41531	{1.30891, 1.4744}	7.51864	ar	61 783.	57 005.2	{56 101., 57 732.}	7.73318
36	R0	{1.65068, 4.91307}	{2.80321, 13.1443}	{{2.64721, 11.9965}, {2.96307, 14.3219}}	{69.8217, 167.537}	inf	1136.	2876.59	{428., 6138.}	182.528	0.449963	newly	2506.	2343.04	{332., 4555.}	61.9842	0.387999	Reff	1.60096	2.69107	{2.4484, 2.93377}	68.0909	ar	66 453.	92 526.9	{90 910., 93 896.}	39.2366
37	R0	{1.46131, 3.06714}	{1.43965, 2.60601}	{{1.43965, 2.60601}, {1.43965, 2.60601}}	{1.48223, 15.0345}	inf	856.	2609.44	{497., 4626.}	225.355	0.50269	newly	3280.	8901.4	{2331., 14 057.}	187.177	0.453598	Reff	1.41952	1.28693	{1.15447, 1.41673}	9.4645	ar	56 452.	54 202.8	{53 240., 55 085.}	3.9842
38	R0	{1.71034, 5.2546}	{2.22453, 8.50182}	{{2.19024, 8.25876}, {2.25463, 8.72462}}	{30.0634, 61.7978}	inf	2107.	4175.22	{520., 11 097.}	133.923	0.397407	newly	4350.	4875.87	{731., 10 690.}	66.5128	0.342225	Reff	1.62197	2.0518	{1.7673, 2.22627}	26.7288	ar	69 816.	84 932.8	{83 907., 85 960.}	21.6524
39	R0	{1.43565, 5.57989}	{1.47488, 4.06661}	{{1.47488, 4.06661}, {1.47488, 4.06661}}	{2.73256, 27.1202}	inf	2976.	4650.19	{2765., 5832.}	64.1452	0.220557	newly	4327.	7250.65	{4578., 9464.}	73.6671	0.236648	Reff	1.28285	0.921633	{0.774135, 1.19266}	29.3642	ar	54 407.	56 717.4	{55 905., 57 514.}	4.24648
40	R0	{1.40327, 4.00764}	{1.31246, 2.23442}	{{1.31246, 2.23442}, {1.31246, 2.23442}}	{6.47131, 44.246}	inf	1552.	2167.4	{200., 3054.}	67.8157	0.291947	newly	3528.	7046.05	{935., 9363.}	114.8	0.356012	Reff	1.32289	1.11086	{0.922095, 1.26866}	16.028	ar	51 976.	43 197.6	{42 241., 44 128.}	16.8894
41	R0	{1.63612, 4.26741}	{1.51531, 2.72346}	{{1.51531, 2.72346}, {1.51531, 2.72346}}	{7.38393, 36.18}	inf	1646.	4026.13	{1755., 6252.}	149.198	0.363301	newly	4452.	12 828.1	{7281., 16 453.}	188.142	0.43923	Reff	1.56264	1.27752	{1.04059, 1.42716}	18.2464	ar	65 029.	59 243.	{58 432., 59 864.}	8.89752
42	R0	{1.57426, 6.50271}	{2.82684, 18.2744}	{{2.68803, 17.202}, {2.89334, 18.7697}}	{79.5661, 181.027}	inf	3056.	8724.3	{1421., 17 569.}	208.024	0.498033	newly	4138.	4163.38	{729., 8181.}	57.8207	0.364144	Reff	1.43171	2.4006	{1.96927, 2.75828}	67.6732	ar	62 522.	92 561.1	{91 295., 93 330.}	48.0456
43	R0	{1.37818, 5.37636}	{1.85533, 9.28761}	{{1.85533, 9.28761}, {1.85533, 9.28761}}	{34.6217, 72.749}	inf	2468.	7820.62	{2520., 12 419.}	223.524	0.476602	newly	3812.	6644.71	{2559., 9452.}	89.2058	0.2882	Reff	1.24355	1.45415	{1.09943, 1.75021}	21.0702	ar	50 098.	75 291.3	{74 879., 75 728.}	50.2881
44	R0	{1.66606, 4.92641}	{2.06604, 6.96677}	{{2.05986, 6.93093}, {2.05986, 6.93093}}	{24.0075, 41.4168}	inf	1205.	3162.6	{648., 7037.}	187.677	0.415386	newly	2554.	4786.77	{1088., 10 216.}	119.539	0.351563	Reff	1.61485	1.93034	{1.76561, 2.03448}	19.6032	ar	67 066.	81 290.5	{80 857., 81 609.}	21.2098
45	R0	{1.623, 5.9449}	{1.43579, 3.38828}	{{1.43579, 3.38828}, {1.43579, 3.38828}}	{11.5348, 43.0053}	inf	2171.	3843.61	{1482., 5168.}	93.0967	0.344392	newly	3513.	8525.35	{4354., 10 696.}	151.994	0.432255	Reff	1.52557	1.15767	{1.00245, 1.355}	24.1158	ar	64 554.	53 839.5	{53 200., 54 612.}	16.5977
46	R0	{1.65235, 5.51234}	{2.58092, 13.7736}	{{2.58092, 13.7736}, {2.58092, 13.7736}}	{56.1969, 149.868}	inf	1774.	3614.59	{400., 7984.}	138.549	0.451817	newly	3211.	2562.49	{305., 5406.}	52.7304	0.412295	Reff	1.57561	2.42327	{2.22553, 2.56223}	53.7985	ar	66 509.	90 300.2	{90 086., 90 511.}	35.7714
47	R0	{1.62138, 8.82208}	{1.75445, 7.55154}	{{1.75445, 7.55154}, {1.75445, 7.55154}}	{8.20721, 14.4018}	inf	5092.	9224.68	{4997., 11 083.}	89.4794	0.28513	newly	4726.	8742.9	{5842., 10 209.}	90.3404	0.283318	Reff	1.36283	1.15187	{0.878418, 1.53325}	20.8844	ar	65 598.	71 490.9	{70 900., 71 883.}	8.98339
48	R0	{1.64073, 6.69261}	{1.53501, 4.02511}	{{1.53501, 4.02511}, {1.53501, 4.02511}}	{6.44347, 39.8574}	inf	3000.	4837.77	{1813., 6819.}	79.5753	0.284474	newly	4095.	9479.	{4690., 12 053.}	138.013	0.379013	Reff	1.5074	1.23994	{1.08691, 1.44363}	17.8195	ar	66 438.	60 554.2	{60 065., 61 260.}	8.85609
49	R0	{1.65556, 3.796}	{1.93145, 4.79243}	{{1.88643, 4.56746}, {1.88643, 4.56746}}	{17.7077, 28.7575}	inf	673.	1746.57	{190., 4013.}	192.416	0.471183	newly	2370.	4577.59	{552., 8665.}	133.716	0.425661	Reff	1.6296	1.91563	{1.71559, 2.43665}	18.2946	ar	66 575.	77 962.6	{75 558., 89 939.}	17.914
50	R0	{1.56828, 3.65713}	{1.82289, 4.40955}	{{1.82289, 4.40955}, {1.82289, 4.40955}}	{16.235, 20.5741}	inf	1346.	3846.42	{880., 7542.}	203.792	0.455273	newly	4132.	9196.89	{2490., 16 252.}	140.752	0.381696	Reff	1.50552	1.65169	{1.4827, 1.78764}	11.3552	ar	62 761.	74 065.1	{73 640., 74 632.}	18.0113
